# A combination of *Citrus aurantifolia* fruit rind and *Theobroma cacao* seed extracts supplementation enhances metabolic rates in overweight subjects: a randomized, placebo-controlled, cross-over study

**DOI:** 10.29219/fnr.v68.10745

**Published:** 2024-08-01

**Authors:** Nihal Kumar Reddy Ammatalli, Sesha Sai Siva Krishna Kuricheti, Sudipta Veeramachaneni, Yean Kyoung Koo, Guru Ramanathan, Amulya Yalamanchi

**Affiliations:** 1 Department of General Medicine, Anu Hospital, Suryaraopet, Vijayawada, India; 2 SV Scientific, Pittsburgh, PA, USA; 3 Department of R&I Center, COSMAXBIO, Seongnam, Republic of Korea; 4 Pharmacology-Based Clinical Trials Laboratory, Pennington Biomedical Research Center, Baton Rouge, LA, USA; 5 Department of General Medicine, Yalamanchi Hospitals and Research Centre, Vijayawada, India

**Keywords:** body fat mass, Citrus aurantifolia, fat and carbohydrate oxidation, resting energy expenditure, thermogenic botanical composition, Theobroma cacao

## Abstract

**Background and objective:**

LN19183 is a proprietary, synergistic combination of *Citrus aurantifolia* fruit rind and *Theobroma cacao* seed extracts that increased resting energy expenditure (REE) in high-fat diet (HFD)-fed obese rats. The objective of this study was to validate the thermogenic potential of LN19183 in obese Sprague Dawley (SD) rats and to assess its clinical efficacy in a proof-of-concept, randomized, placebo-controlled, cross-over human trial.

**Methods:**

In the rat study, HFD-fed obese rats were supplemented with either HFD alone or with 45, 90, or 180 mg LN19183 per kg body weight (BW) for 28 days. In the human study, 60 overweight adults (male and female, aged 20–39 years) were randomized. Subjects took LN19183 (450 mg) or a matched placebo capsule on two consecutive days in phases one and two of the study, separated by a 10-day washout period. In each phase, on day 1, REE at pre-dose, 60-, 120-, and 180-min post-dose, and on day 2, metabolic rates at pre-dose and post-dose during and 20 min after exercise were measured using indirect calorimetry.

**Results:**

In rats, LN19183 significantly increased REE, reduced BW gain and fat masses, and increased fat and carbohydrate metabolism marker proteins including beta 3 adrenergic receptor (β3-AR), phospho-AMP-activated protein kinase (AMPK), glucagon-like peptide-1 receptor (GLP-1R) in the liver, and serum adiponectin levels. Furthermore, LN19183-supplemented human volunteers increased (*P* < 0.05, vs. placebo) the metabolic rates at rest and with exercise; their fat oxidation was increased (*P* < 0.05, vs. placebo) at rest and 20 min post-exercise. The groups’ systolic and diastolic blood pressure (BP), heart rates (HR), and safety parameters were comparable.

**Conclusion:**

These observations suggest that LN19183 is a thermogenic botanical composition with no stimulatory effects on BP and HR.

## Popular scientific summary

- LN19183 is a proprietary, synergistic thermogenic combination containing *Citrus aurantifolia* fruit rind and *Theobroma cacao* seed extracts.
- In HFD-fed obese rats, LN19183 significantly increased resting energy expenditure, reduced adiposity, and increased fat and carbohydrate metabolism marker proteins.
- In a human study, LN19183 significantly increased the metabolic rates at rest and, with exercise, increased fat and carbohydrate oxidations.
- The participants’ hemodynamic variables (heart rate and blood pressure) were within the normal ranges.

Obesity has become a leading health problem worldwide, which threatens both psychosocial and physical health, contributing to a higher occurrence of many chronic complications (1, 2). It is often associated with comorbidities, including cardiovascular dysfunctions, type 2 diabetes, and hypertension, also significantly lowering life span (3). Reduced energy expenditure contributes to a pivotal role in the development of obesity through positive energy balance, resulting in increased adiposity and weight gain (4, 5). In most weight loss programs, major emphasis is given to manipulating diet, appetite, and exercise. Besides, the FDA has also approved pharmaceutical drugs such as Somatotropin or Setmelanotide as an effective strategy for the treatment of obesity by increasing energy expenditure (6, 7).

Fibroblast growth factor-21 (FGF-21) has emerged as a potential therapeutic target for metabolic dysfunctions since the administration of FGF-21 causes improved glycemic control, lipid profile, and mainly weight loss by the elevation of energy expenditure in mice and rodents (8–10). The FGF-21-mediated elevation of energy expenditure has been attributed to the induction of mitochondrial uncoupling protein 1 (UCP-1) (11). The transformation of white adipose tissue (WAT) to brown adipose tissue (BAT) is characterized by the increased expression of UCP-1 and is reported as the basis of adaptive thermogenesis in adipose tissue (11, 12). Recently, in a proof-of-concept *in vitro* and *in vivo* study, Kundimi et al. have demonstrated that a proprietary composition (LN19183) containing aqueous-ethanol extracts of *Citrus aurantifolia* fruit rind and *Theobroma cacao* seeds synergistically enhanced FGF-21 production in 3T3-L1 adipocytes. LN19183 was more potent than the individual extracts and the most effective among various combinations of *C. aurantifolia* and *T. cacao* extracts tested *in vitro*. Furthermore, LN19183 supplementation increases UCP-1 protein expression in inguinal fat tissue and enhances resting energy expenditure (REE) and fat oxidation of HFD-fed obese rats via activation of FGF-21/β3-AR/UCP-1 axis (13).

*Citrus aurantifolia* bioflavonoids, including naringenin and nobiletin, have been shown to reduce cardiovascular risk factors such as blood pressure, obesity, and dyslipidemia (14, 15). Citrus fruits are rich in hesperidin and are reported to exert a lipid-lowering effect by regulating lipid and glucose metabolism via AMPK and PPAR signaling pathways (16, 17). *Theobroma cacao* is well known for its antioxidant and antitumor efficacies (18, 19). Cocoa flavan-3-ols have been shown to attenuate hypercaloric diet-induced obesity through enhanced β-oxidation and energy expenditure in white adipose tissue (20). Theobromine is one of the abundant methylxanthines in cocoa. Jang et al. reported the inhibitory effects of theobromine on differentiation and adipogenesis in 3T3-L1 adipocytes via activation of AMPK and suppression of the ERK and JNK signaling pathways (21).

The present investigation confirms the efficacy of the botanical blend LN19183 in increasing REE, preventing body weight and fat gain in HFD-fed obese rats, and generates proof-of-concept data on increased energy expenditure in LN19183-supplemented overweight male and female volunteers at rest and with exercise in a double-blind, placebo-controlled, cross-over clinical trial.

## Materials and methods

### Reagents and chemicals

Pierce BCA protein assay kit (cat# 23225), UCP-1 antibody (cat# PA1-24894), Superblock (cat# 37515), and Super Signal West Pico PLUS chemiluminescent Western blot substrate (cat# 34580) were obtained from Thermo-Fisher Scientific, Waltham, MA. Antibodies specific to phospho-AMPKα (Thr172) (cat# 2531S), AMPKα (cat# 2532S), Acetyl-CoA carboxylase (ACC; cat# 3676S), Phospho-Acetyl-CoA carboxylase (Ser79) (Phospho-ACC; cat# 3661S), and Glyceraldehyde 3-phosphate dehydrogenase (GAPDH, clone 14C10) rabbit mAb (cat# 2118S) were procured from Cell Signaling Technology, Danvers, MA. Glucagon-like peptide-1 receptor (GLP-1R) antibody (cat# 26196-1-AP) was purchased from Proteintech, Rosemont, IL; β3-AR antibody (cat# orb221343) was procured from Biorbyt, Cambridge, UK; and HRP-conjugated goat anti-rabbit IgG (H + L) (cat#111–035-003) or goat anti-mouse IgG (H + L) (cat# 115–035-003) were purchased from Jackson Immunoresearch, West Grove, PA.

### Study material

A standardized, proprietary composition LN19183 containing aqueous-ethanol (50%) extracts of *C. aurantifolia* fruit rind (CA) and water extract of *T. cacao* seed (TC) blended at a 2:1 ratio is produced in a cGMP-certified facility at Laila Nutraceuticals, Vijayawada, India. The plant raw material collection, extraction procedure, and standardization are described earlier (13). LN19183 is standardized to contain at least 5.0% citric acid, 0.5% theobromine, and 0.3% hesperidin by the high-performance liquid chromatography (HPLC) method. The final composition, LN19183, is also known as CL19183 or TheoLim™.

## In vivo *study*

### Animal care and husbandry

Male Sprague Dawley rats (body weight [BW] 255.6–430.1 g; aged 12–14 weeks) were procured from Mahaveera Enterprises, Hyderabad, Telangana, India, and acclimatized for seven days under the standard laboratory conditions (23 ± 2 °C; relative humidity: 40–70%; 12-h light/dark cycle). The rats were fed a standard pellet diet (cat# 1324, ATNT Laboratories, and Mumbai, India) and reverse osmosis (RO) water *ad libitum*. Institutional Animal Ethics Committee (IAEC), Laila Impex, Vijayawada, India, authorized the research protocol (Protocol No.: LI/IAEC/ LI211012). The animal care and experimental techniques were followed according to the guidelines of the Committee for the Purpose of Control and Supervision of Experiments on Animals (CPCSEA), India.

### Experimental design

The animal experiment followed a similar protocol described earlier with some modifications (13). This study had two phases: the induction phase and the supplementation phase. In the 42-day induction phase, 8 animals received the regular rodent pellet diet (RD; cat# 1324, ATNT Laboratories, and Mumbai, India) (G1; n = 8) and 32 animals received a high-fat-containing diet (HFD- 45% kcal fat; cat# D12451, Research Diet Inc., New Brunswick, NJ) *ad libitum*. On day 43, the HFD-fed rats were randomly divided into four groups (n = 8); G2, G3, G4, and G5 received HFD only and HFD plus 45, 90, or 180 mg/kg BW LN19183 daily, respectively, through oral gavage with 0.5% (w/v) carboxymethylcellulose as the vehicle. The treatment or supplementation phase lasted for 28 days. The G1 rats received the regular diet through the induction and supplementation periods. Each gram of RD and HFD provided 3.23 and 4.73 kcal energy, respectively. The rats’ body weights were measured weekly using an electronic balance (Sartorius, Model# GE7101, Göttingen, Germany). The resting energy expenditure (REE), clinical biochemistry, serum markers, and histopathological assessments were performed at the end of the supplementation phase.

### Resting energy expenditure

The REE of the experimental rats was measured as described earlier using an indirect calorimetric system with an airtight four-chamber metabolic cart (Model# LE1332, Oxylet Pro 4, Panlab, Spain) equipped with a gas analyzer (cat# LE405, Harvard Apparatus, Cornella, Spain) (13). Briefly, rats were acclimatized within the metabolic chamber for 2 h before measuring O_2_ and CO_2_ exchange using the pre-calibrated gas analyzer. The volumes of O_2_ and CO_2_ exchanges were recorded at one-minute intervals and analyzed for 4 h (METABOLISM V2.2.01, Harvard Apparatus, Cornella, Spain). The captured O_2_ and CO_2_ data were normalized to the rats’ body weights and presented as VO_2_ and VCO_2_ (mL/min/kg). REE (kcal/kg BW/day) was calculated using the modified Weir equation as described earlier (13). REE was calculated using the formula: REE (kcal/kg/day) = [3.941 (VO_2_) + 1.106 (VCO_2_)] × 1,440.

### Necropsy

On the final day of the supplementation period, after CO_2_ euthanasia, adipose tissues (abdominal, inguinal, epididymal, mesenteric, and interscapular) and the liver were collected from the experimental rats. The tissue weights were measured using an analytical balance (Model# CP224S; Sartorius, Göttingen, Germany). The tissues were stored at -80°C in aliquots for biochemical and molecular markers analyses.

### Tissue lysates preparation

The inguinal fat or liver tissue samples were lysed in CelLytic™ MT cell lysis reagent (cat# C3228; Sigma-Aldrich, St. Louis, MO) containing a protease and phosphatase inhibitor cocktail (cat# P5726; Sigma Aldrich, St. Louis, MO). The protein content in the clarified tissue lysates (14,000 × g, 10 min at 4°C) was quantified using the BCA protein assay kit (cat# 23225; Thermo Scientific, Waltham, MA).

### Serum biochemical and biomarker analysis

The serum was separated (3,200 × g) from the blood samples collected by puncturing the retro-orbital plexus from overnight fasted rats under mild anesthesia. Serum levels of aspartate aminotransferase (AST), alanine aminotransferase (ALT), creatinine, urea, blood urea nitrogen (BUN), total cholesterol (TC), triglycerides (TG), and glucose were measured using a clinical chemistry autoanalyzer (Mindray BS-240; Shenzhen, China).

Serum adiponectin (cat# EZRADP-62K, Merck-Millipore, Burlington, MA), leptin (cat# EZRL-83K, Merck-Millipore, Burlington, MA), and insulin (cat# 80-INSRT-E01, ALPCO, Boston, MA) levels were measured using commercially available ELISA kits following the manufacturer’s protocols. The liver tissue lysates were analyzed for liver triglycerides (TG) using the colorimetric assay kit (cat# 10010303, Cayman Chemical, Ann Arbor, MI). The analytical sensitivities for adiponectin, leptin, insulin, and TG kits were 0.4 ng/mL, 0.04 ng/mL, 0.124 ng/mL, and 0.5 mg/dL, respectively.

### Fat cell morphometry

At terminal necropsy, the excised epididymal fat tissues were fixed in 10% neutral buffered formalin for 48 h at room temperature and then embedded in paraffin. The paraffin-embedded tissues were sectioned at 5 μm thickness using a rotating microtome (Cat# RTS2125, Leica Biosystems, Wetzlar, Germany). The hematoxylin-eosin-stained tissue sections were examined at 200X magnification under a light microscope (Zeiss Axio Scope A1, Carl Zeiss GmbH, Jena, Germany), and the images were captured using a CCD camera (ProgRes C5 from Gen Optik. Jena, Germany). At least 200 fat cells were randomly selected from each tissue section, and the cross-sectional area was measured using Adiposoft 1.16 (Image J, NIH, Bethesda, MD).

### Western blot assay

The western blot assay was performed as described earlier (13). An equal amount of the liver or fat tissue lysate proteins was resolved on 10% sodium dodecyl sulfate-polyacrylamide gel electrophoresis (SDS-PAGE). The resolved proteins were blotted onto nitrocellulose (NC) membrane, blocked the non-specific sites, and incubated with the primary antibodies (dilution 1:1,000) overnight at 4°C. After the incubation period, the washed membranes were probed with respective secondary antibodies at 1:10,000 dilutions for 45 min. The specific protein bands were developed using a chemiluminescent substrate (Super Signal West Pico PLUS). The stripped NC membranes were reprobed with GAPDH antibodies and developed to ensure equal protein loading. The protein bands were captured in a ChemiDoc MP imager (Bio-Rad, Hercules, CA), and the protein expressions were measured using molecular imaging software v.5.0.2.30 (Carestream Health, Rochester, NY).

## Clinical study

### Ethical approval and subject recruitment

The institutional ethics committee of Anu Hospitals, Vijayawada, Andhra Pradesh, India (ECR/1049/Inst/AP/2018/RR-21), approved the study protocol**.** The approved protocol was registered in the Clinical Trial Registry of India (CTRI/2021/04/032478) on April 01, 2021. The study was conducted following the ethical principles of the Declaration of Helsinki and was consistent with Good Clinical Practice and the applicable regulatory requirements.

After voluntarily signing the informed consent form, male and female participants were screened for enrollment into the study based on inclusion and exclusion criteria (Supplementary Table 1). A total of 60 healthy male and female subjects (aged: 20–39 years; body mass index [BMI]: 25–29.9 kg/m^2^) volunteered to participate in this randomized, double-blind, placebo-controlled, cross-over study. The subjects followed their regular dietary pattern during the study. Fat, protein, carbohydrate, and total calorie consumption were recorded and monitored using a dietary calculation software, DietSoft (Invincible Ideas, Noida, Uttar Pradesh, India) (22). The subjects were advised to maintain daily diaries and instructed to take one capsule of 450 mg LN19183 before breakfast on the assessment days. The study coordinators and the principal investigator monitored and endorsed the daily diaries.

### Study design

The present human trial was designed as a randomized, double-blinded, placebo-controlled, cross-over study. The recruited participants were randomly divided into two groups (n = 30): placebo followed by LN19183 and LN19183 followed by placebo. The participants were allocated to either placebo or LN19183 group (1:1) based on the randomization codes generated by a block design using the SAS procedure PROC PLAN. The participants were recruited in groups according to the order of enrollment. The investigators and the participants were unaware of the randomization code and allocation detail until all evaluations were completed at the end of the study.

Each participant took one identical capsule containing either 450 mg LN19183 or a matched placebo before breakfast on days 1 and 2 of the trial (phase 1). After a 10-day washout period, the participants were crossed over, and a similar intervention was followed on days 13 and 14 of the study (days 1 and 2 of phase 2). On day 1 of phases 1 and 2, resting metabolic rate (RMR) was measured at pre-dose (0 min) and 60, 120, and 180 min post-dose using indirect calorimetry. On day 2 of phases 1 and 2, the metabolic rate (MR) was measured at pre-dose, 1 h post-dose during exercise performed on a bicycle ergometer (Wattbike AtomX, Nottingham, UK), and 20 min after exercise.

### Efficacy evaluations

The participants rested in comfortable sitting positions for 5 min. Participants’ resting heart rate (HR) and blood pressure (BP) were recorded using an automated, oscillometric blood pressure monitor (Omron 5 series Model BP742, Lake Forest, IL). The average of three readings of HR and BP was recorded.

For RMR measurements, the participants were rested in a reclined position for 10 min. RMR was measured using a canopy hood attached to a human metabolic cart (Iworx Systems Inc, Dover, NH). The device measured the metabolic rate at rest and during exercise utilizing the respiratory exchange ratio (RER) from the volume of CO_2_ (VCO_2_) produced divided by the consumed volume of oxygen (VO_2_). On day 2, the participants wore a face mask attached to the metabolic cart to capture the VCO_2_ and VO_2_ (L/min). Fat oxidation and carbohydrate oxidation were estimated using the following formula, considering minimum protein utilization (23).

Fat oxidation = (1.695 X VO_2_) – (1.701 X VCO_2_)

Carbohydrate oxidation = (4.585 X VCO_2_) – (3.226 X VO_2_)

The psychometric evaluation to assess the mood disturbance was performed using a short form of the Profile of Mood States (POMS-SF) questionnaire on days 1 and 2 of both phases of the study. Also, the serum free fatty acid (FFA) levels were measured in days 1 and 2 fasting blood samples using an FFA quantification kit (cat# K612-100, Biovision, Waltham, MA) following the user’s protocol. The sensitivity of this colorimetric assay kit is 2 μM.

### Safety assessments

The safety assessments included monitoring the adverse events, vital signs, and measurements of clinical laboratory parameters (serum biochemistry, hematology, and urine analysis). In serum biochemistry, fasting glucose, alanine transaminase (ALT), aspartate aminotransferase (AST), alkaline phosphatase (ALP), creatinine, uric acid, blood urea nitrogen (BUN), bilirubin, albumin, sodium, and potassium were estimated; in hematology, red blood cell and platelets count, hemoglobin, total leukocytes, and differential count, and erythrocyte sedimentation rate (ESR) were estimated; and in routine urine analysis, pH, color, specific gravity, protein, glucose, and RBC were performed, at screening and the end of the trial (day 14).

### Statistical analysis

The data are presented as the mean ± standard deviation (SD). In the rat study, RD vs. HFD and HFD vs. LN19183-supplemented groups comparison analyses were performed using ANOVA, respectively, with suitable *post-hoc* tests using GraphPad Prism software v6.01 (GraphPad Software, Inc., San Diego, CA, USA).

In the present cross-over human study, 60 subjects’ data (n = 30; combining phases 1 and 2) analysis provided 90% power with a 95% confidence interval (CI) by anticipating a mean difference (d) of 52 kcal/day (24) and an average SD of 59, assuming a 10% dropout. Data were analyzed to compare the mean differences within the group using paired *t*-tests and mixed effect model for differences between the groups after meeting the normality assumptions in the Shapiro-Wilk test using SAS version 9.4 for Windows. A two-sided *P*-value of < 0.05 was considered statistically significant.

## Results

### In vivo study

#### LN19183 supplementation reduced body weight gain and fat mass and increased resting energy expenditure (REE) in HFD-fed obese rats

**Fig. 1:**
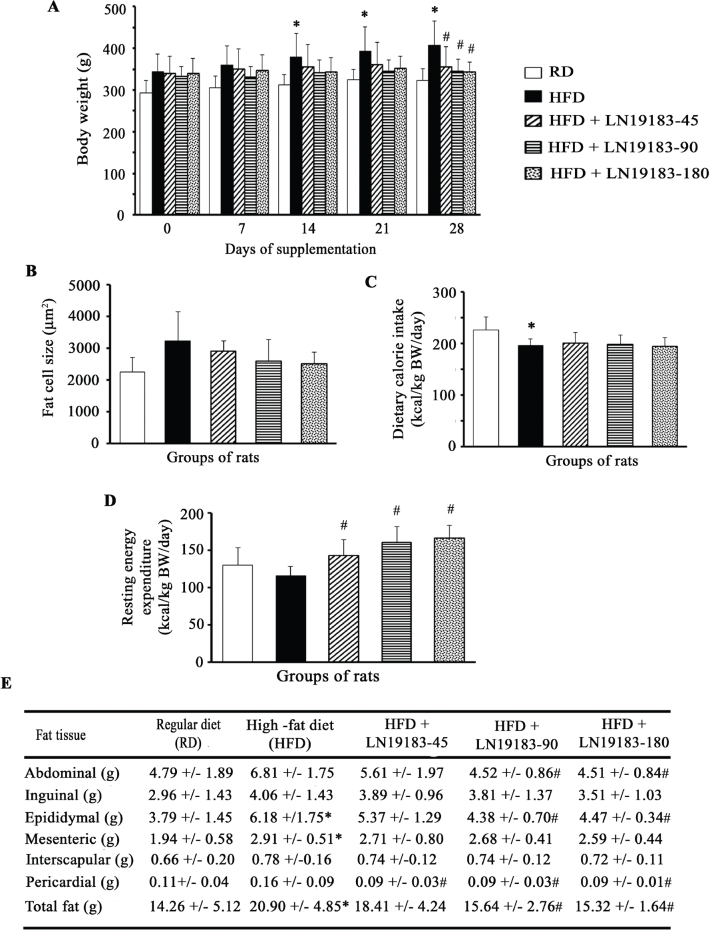
**(A)** The bar graph shows changes in body weights of the experimental rats. Body weights (BW) are measured weekly. The rats received either a regular diet (RD) or a high-fat diet (HFD) or HFD in combination with a daily dose of 45 mg/kg (LN19183-45), 90 mg/kg (LN19183-90), or 180 mg/kg (LN19183-180) of LN19183, as described in the Materials and Methods. **(B)** Each bar presents the mean ± SD of area (μm^2^) of the epididymal fat cells in the experimental groups of rats. The bar graphs **(C)** and **(D)** present daily dietary calorie intake (kcal per kg BW) and resting energy expenditure (kcal per kg BW) by the groups of rats as indicated. **(E)** The table presents the total body fat and regional fat masses in the rats of the experimental groups. Data present as mean ± SD; n = 8. *, and # indicate significance (*P* < 0.05) in RD vs. HFD, and HFD vs. LN19183-supplemented rats, respectively, using ANOVA with suitable *post-hoc* test.

The bar diagram (Fig. 1A) presents body weight growth patterns and dose-dependent decreases in body weights of LN19183-supplemented obese rats during the study. The increases in body weight in the HFD group were 17.74% (*P* = 0.2490), 21.63% (*P* = 0.0029), 21.45% (*P* = 0.0019), and 26.18% (*P* = 0.0001) on days 7, 14, 21, and 28, respectively, as compared to the rats on a regular diet (RD). The low, mid, and high doses of LN19183 supplementation reduced the body weights by 12.56% (*P* = 0.0383), 15.35% (*P* = 0.0065), and 15.92% (*P* = 0.0043) on day 28, respectively, in comparison with the HFD rats. At the end of the 28-day supplementation period, the body weight gain in HFD rats was 18.41%; in contrast, the low, mid, and high doses of LN19183-supplemented rats gained the body weight by 5.10%, 3.54%, and 0.97%, respectively, from the start of supplementation (Fig. 1A).

The fat cell morphometry analyses reveal that the average epididymal fat cell size was increased by 43.61% (*P* = 0.0532; vs. RD) in HFD rats; the cell size in 45, 90, and 180 mg doses of LN19183-supplemented rats was reduced by 9.54% (*P* = 0.4305), 19.65% (*P* = 0.1675), and 22.03% (*P* = 0.1141), respectively, as compared to the HFD rats (Fig. 1B).

During the 28-day supplement duration, the daily dietary calorie intake by the HFD rats was significantly lower in comparison with the RD rats; however, the daily calorie intake by the LN19183-supplemented rats was not significantly different as compared to the HFD rats (Fig. 1C).

The REE in LN19183-supplemented rats was dose-dependently elevated by 23.26% (*P* = 0.0259), 38.38% (*P* = 0.0005), and 43.64% (*P* = 0.0001) in the low-, mid-, and high-dose groups, respectively, as compared to the HFD rats (Fig. 1D).

The effects of LN19183 supplementation in reducing total and regional fat masses in obese rats are tabulated in Fig. 1E. At termination, the HFD rats gained total body fat mass by 46.59% (*P* = 0.0421) compared to the RD-fed rats (Fig. 1E). The 45, 90, and 180 mg/day doses of LN19183-supplemented rats reduced 11.91% (*P* = 0.2832), 25.13% (*P* = 0.0350), and 26.67% (*P* = 0.0097) total fat masses, respectively, as compared to the HFD-fed rats (Fig. 1E). In mid- and high-dose LN19183-supplemented rats, abdominal, epididymal, and pericardial fats are significantly reduced compared to the HFD-fed rats (Fig. 1E).

#### LN19183 supplementation modulated metabolic markers and fat metabolism-related endocrine factors in the serum of obese rats

The serum levels of fat metabolites, liver transaminases, and glucose are elevated in the HFD-fed obese rats. LN19183 supplementation reduced the elevated levels of these metabolic markers in the rats. However, compared with the HFD rats, the changes in the serum levels of these measures are not significant. Also, the serum BUN, urea, and creatinine levels in the LN19183-supplemented rats were reduced, but the changes were not significant compared to the HFD and RD-fed rats (Table 1).

**Table 1:** Serum levels of metabolic and endocrine markers in LN19183-supplemented obese rats

Parameters	Regular diet (RD)	High-fat diet (HFD)	HFD + LN19183-45	HFD + LN19183-90	HFD + LN19183-180
Glucose (mg/dL)	67.25 ± 17.00	74.63 ± 15.54	70.50 ± 12.01	68.88 ± 10.89	66. 63 ± 12.97
Total cholesterol (mg/dL)	48.88 ± 5.14	61.25 ± 10.25*	56.25 ± 7.32	51.88 ± 6.13#	45.38 ± 6.16#
Triglycerides (mg/dL)	44.38 ± 17.09	66.75 ± 12.19*	51.75 ± 14.78	50.71 ± 9.73	48.50 ± 15.76
AST (U/L)	169.13 ± 37.36	173.88 ± 45.19	138.14 ± 19.84	138.00 ± 24.55	136.63 ± 18.42
ALT (U/L)	66.25 ± 19.70	73.25 ± 21.53	59.13 ± 15.02	56.00 ± 13.42	57.13 ± 8.61
BUN (mg/dL)	17.38 ± 4.53	15.13 ± 2.36	15.00 ± 1.85	14.75 ± 3.85	13.75 ± 2.12
Urea (mg/dL)	37.25 ± 9.48	32.25 ± 5.06	32.13 ± 4.29	31.50 ± 8.12	29.50 ± 4.69
Creatinine (mg/dL)	0.40 ± 0.06	0.41 ± 0.04	0.39 ± 0.04	0.36 ± 0.03	0.38 ± 0.04
Adiponectin (μg/mL)	15.93 ± 2.20	12.13 ± 1.43*	13.78 ± 1.48	14.54 ± 0.94#	14.64 ± 2.27#
Leptin (ng/mL)	2.33 ± 1.06	5.26 ± 1.92*	3.66 ± 1.39	3.30 ± 1.72	3.22 ± 1.68#

Data represent mean ± SD; n = 8.

* and

# indicate significance (*P* < 0.05) in RD vs.

HFD-supplemented group, and HFD vs. LN19183-supplemented group, respectively, using one-way ANOVA followed by Dunnett’s *post-hoc* test.

In the HFD rats, the serum adiponectin levels reduced significantly by 23.85% (*P* = 0.0024), compared to the RD rats (Table 1). LN19183 supplementation showed dose-dependent recovery with 19.87% (*P* = 0.0487, vs. HFD) and 20.69% (*P* = 0.0381, vs. HFD) in adiponectin levels in the 90 and 180 mg groups, respectively. The leptin levels significantly increased in the HFD rats (125.75%; *P* = 0.0154) compared to the RD rats. The low, mid, and high doses of LN19183 supplementation gradually reduced the serum leptin levels in the obese rats. The LN19183-180 supplemented rats exhibited a 38.78% (*P* = 0.0487) reduction in leptin levels compared to the HFD-fed obese rats (Table 1).

#### LN19183 supplementation upregulated fat and energy metabolism-related marker protein expressions in the liver and inguinal fat tissues of obese rats

**Fig. 2:**
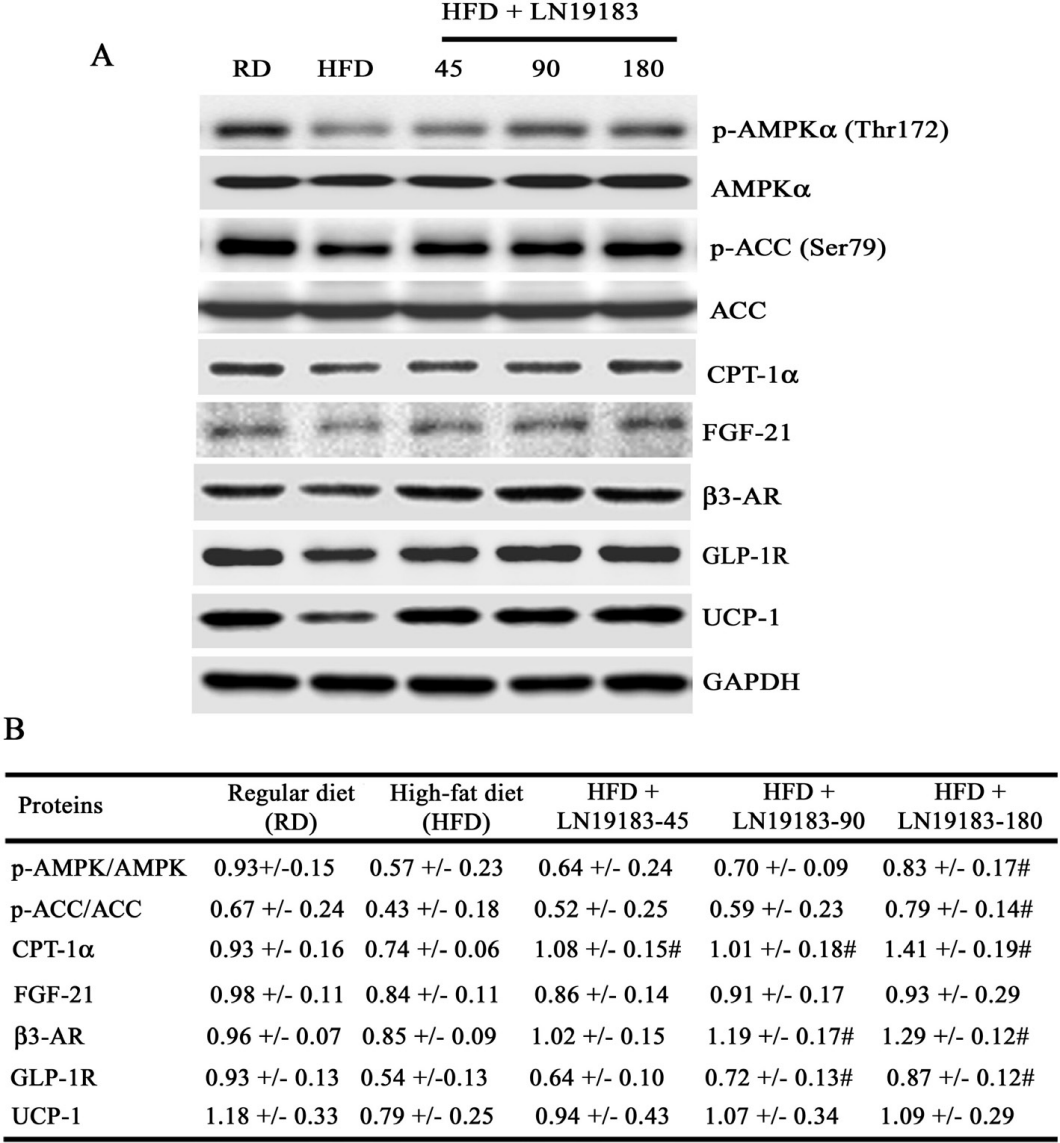
**(A)** Representative immunoblot images showing protein expressions of p-AMPK and AMPK, p-ACC and ACC, CPT-1α, β3-AR, GLP-1R, and FGF-21 in liver and UCP-1 in inguinal fat tissue lysates of the experimental groups of rats as indicated. **(B)** The table presents group-wise mean ± SD (n = 8) of the normalized protein expressions obtained from the densitometry analysis of the immunoblot protein bands. p-AMPK and p-ACC protein expressions were normalized with the respective un-phosphorylated proteins, and CPT-1α, β3-AR, FGF-21, GLP-1R, and UCP-1 were normalized with GAPDH. RD: regular diet, HFD: high-fat diet; # indicates significance (*P* < 0.05) in HFD vs. LN19183-supplemented with 45, 90, or 180 mg/kg/day groups comparison analyses using one-way ANOVA with suitable *post-hoc* test.

Fig. 2A depicts representative immunoblot images showing modulations of key marker proteins that regulate fat and energy metabolism in the experimental rats’ liver and inguinal fat tissues. Fig. 2B summarizes the group-wise data (n = 8; mean ± SD) of the normalized protein expressions obtained from the densitometry analyses of the immunoblot bands. In the HFD-induced obese rat liver tissues, p-AMPK, p-ACC, CPT-1α, FGF-21, β3-AR, and GLP-1R protein expressions were reduced by 38.71%, 35.82%, 20.43%, 14.29%, 11.46%, and 41.94%, respectively, in comparison with the RD-fed rats. In comparison with the HFD rats, the p-AMPK and p-ACC expressions in the LN19183-180 were increased by 45.61% (*P* = 0.0301) and 83.72% (*P* = 0.0070); CPT-1α expressions were increased by 45.95% (*P* = 0.0006), 36.49% (*P* = 0.0069), and 90.54% (*P* = 0.0001) in the 45, 90, and 180 mg LN19183-supplemented groups, respectively (Fig. 2B). The β3-AR expression was increased by 40% (*P* = 0.0001) and 51.76% (*P* = 0.0001) in the 90 and 180 mg LN19183-supplemented groups, respectively, compared to the HFD rats. As compared to the HFD rats, the low-, mid-, and high-dose LN19183-supplemented obese rats showed 18.52% (*P* = 0.5166), 33.33% (*P* = 0.0326), and 61.11% (*P* = 0.0001) increases in the GLP-1R expressions, respectively. The liver FGF-21 expressions were increased by 2.38%, 8.33%, and 10.71%; and the inguinal fat UCP-1 were increased by 18.98%, 35.44%, and 37.97% in the low-, mid-, and high-dose LN19183-fed rats vs. HFD rats, respectively. However, these improvements were not significant (Fig. 2B).

## Clinical study

### Demographics and baseline characteristics of the clinical study subjects

**Fig. 3:**
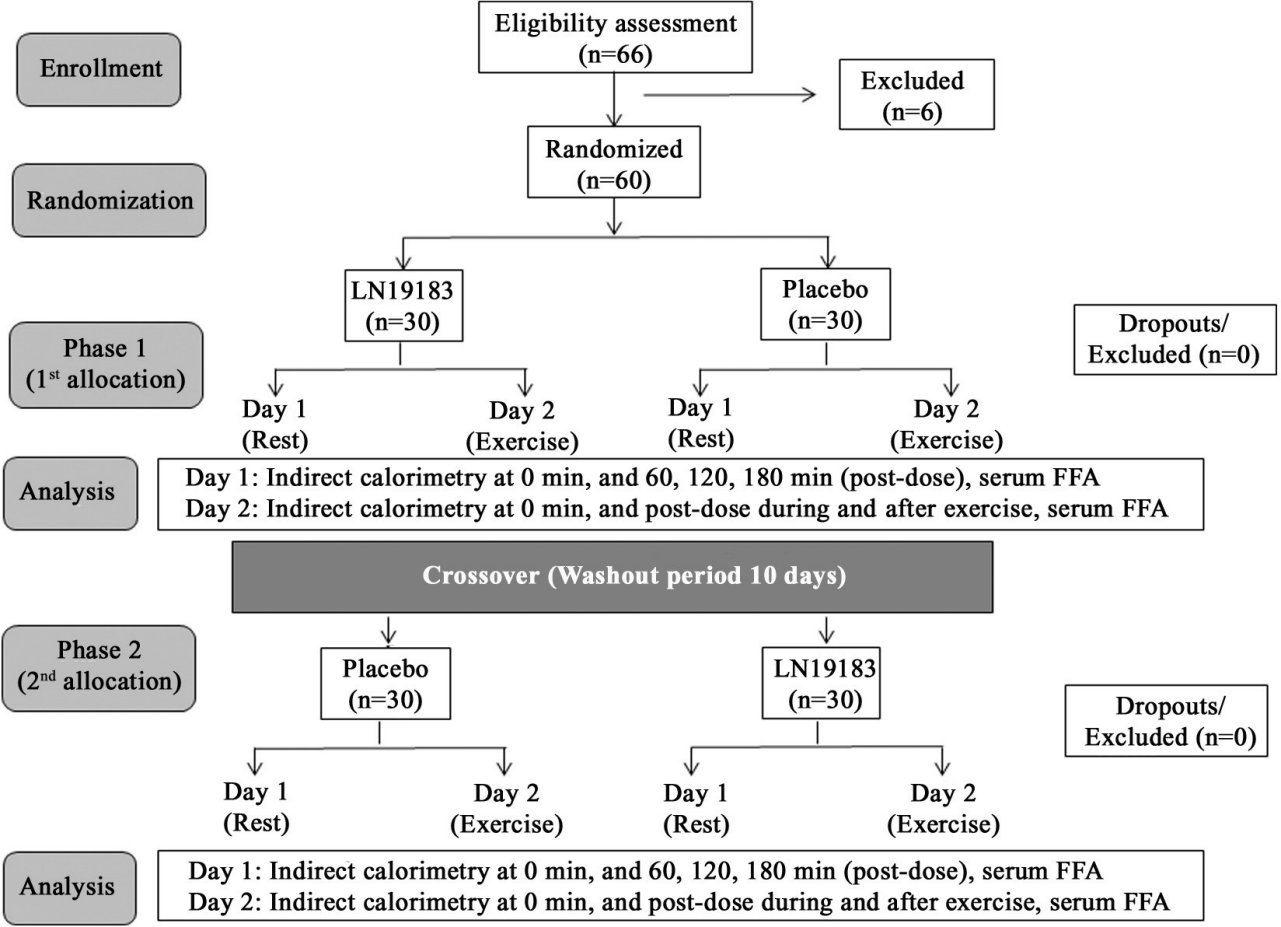
The CONSORT flow diagram presents the participants’ enrollment, randomization, intervention, wash-out period, and analysis of the cross-over trial.

At baseline (day 1 of phase 1), the placebo group (n = 30) contained 18 males and 12 females, and the LN19183 group (n = 30) contained 17 males and 13 females. The mean ± SD of the participants’ age, body weight, and body mass index (BMI) were 27.1 ± 4.2 years, 80.8 ± 3.8 kg, and 26.5 ± 1.0 kg/m^2^ in placebo, and 27.0 ± 3.8 years, 80.5 ± 4.1 kg, and 26.5 ± 1.1 kg/m^2^ were in LN19183 group, respectively. Between-the-groups comparison analysis found that the baseline parameters were not significantly different. The daily average (± SD) of fat, protein, and carbohydrate consumptions (% energy) were 27.36 ± 8.29, 14.11 ± 3.10, and 57.55 ± 8.11 in the placebo and 26.60 ± 9.66, 14.64 ± 3.01, and 58.53 ± 10.51 in the LN19183 group, respectively. The placebo and LN19183 groups consumed 2051.99 ± 629.07 and 2117.65 ± 610.65 kcal/day, respectively. The differences in the macronutrients and total calorie consumptions between the groups were not significant. The participants did not consume any concomitant medication during the study. No participant discontinued the trial or dropped out due to noncompliance with the study. The design and flow of the trial are schematically presented in a diagram following the CONSORT guidelines (Fig. 3).

### LN19183 supplementation increased the metabolic rate at rest and with exercise

**Fig. 4:**
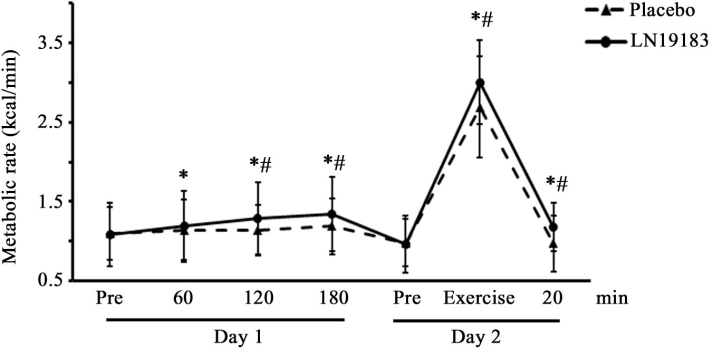
The line diagram shows metabolic rates in resting condition and with exercise in the LN19183 and placebo groups. Data present mean ± SD (n = 60) of metabolic rates (kcal/min) at different time points of the intervention on day 1 (in resting condition) and on day 2 (with exercise) as described in the Materials and Methods. * and # indicate significance (*P* < 0.05) in intragroup comparison (vs. pre-dose) and intergroup comparison (vs. placebo) analyzed using paired *t*-test and a mixed model considering treatment, period, and sequence as fixed factors, respectively.

A line diagram presents the metabolic rates of the placebo and LN19183-supplemented subjects at resting condition (RMR) on day 1 and metabolic rate during and after exercise on day 2 (Fig. 4). A single dose of 450 mg LN19183 gradually increased RMR by 10.19% (*P* = 0.0061), 19.44% (*P* < 0.0001), and 24.07% (*P* < 0.0001) after 60, 120, and 180 min of supplementation, respectively, as compared to the pre-dose. Between-the-groups comparison analysis revealed that the RMR in the LN19183 group was significant at 120 min (*P* = 0.0014) and 180 min (*P* = 0.0002). The placebo group showed a significant increase (8.18%; *P* = 0.0344) in RMR at 180 min from the pre-dose value (Fig. 4).

Similarly, a significant increase in metabolic rate (MR) was observed after a single dose administration of LN19183 during and after 20 min of exercise (212.5%, *P* < 0.0001; 22.91%, *P* < 0.0001 vs. pre-dose, respectively) on day 2 (Fig. 4). In contrast, the placebo group showed 174.49% (*P* < 0.0001) increase and a slight reduction of MR during and 20 min post-exercise, respectively. The MRs in the LN19183 group during (*P* = 0.0033) and post-exercise (*P* < 0.0001) were significantly increased compared to the placebo (Fig. 4).

### LN19183 supplementation enhanced fat and carbohydrate oxidations in the participants

Table 2 summarizes the fat oxidation and carbohydrate oxidation in the participants at rest and with exercise conducted on days 1 and 2, respectively. On day 1, LN19183 increased fat oxidation by 7.92% (*P* = 0.1351), 17.68% (*P* = 0.0012), and 20.91% (*P* = 0.0009), and carbohydrate oxidation by 34.86% (*P* = 0.1202), 46.83% (*P* = 0.0106), and 61.44% (*P* = 0.0051) at 60, 120, and 180 min, respectively, as compared to the pre-dose (Table 2). As compared to the placebo, LN19183 administration significantly increased fat oxidation at 120 min (*P* = 0.0075) and 180 min (*P* = 0.0391) post-dose on day 1, and on day 2, 20-min post-exercise (*P* = 0.0310) (Table 2).

**Table 2:** Calorimetric determination of fat oxidation and carbohydrate oxidation, and free fatty acids levels in the serum of the participants

Interventions	Evaluations	Fat oxidation, mg/min (% change from pre-dose)	Carb. oxidation, mg/min (% change from pre-dose)
Day 1			
Placebo (*n* = 60)	Pre-dose	97.55 ± 45.37	49.50 ± 51.14
	60 min	100.90 ± 41.69 (3.43)	50.30 ± 69.34 (1.61)
	120 min	96.94 ± 41.55 (-0.62)	59.64 ± 58.53 (20.48)
	180 min	101.70 ± 47.85 (4.25)	60.01 ± 77.56 (21.23)
LN19183-450 (*n* = 60)	Pre-dose	94.49 ± 40.08	44.77 ± 63.01
	60 min	101.98 ± 45.99 (7.92)	60.38 ± 63.97 (34.86)
	120 min	111.20 ± 44.86 (17.68)*#	65.74 ± 56.08 (46.83)*
	180 min	114.25 ± 49.90 (20.91)*#	72.28 ± 55.50 (61.44)*
Day 2			
Placebo (*n* = 60)	Pre-dose	94.20 ± 43.13	45.53 ± 65.61
	During exercise	166.23 ± 68.70 (76.46)*	355.17 ± 206.87 (680.07)*
	20 min post-exercise	61.51 ± 34.21 (-34.70)*	89.78 ± 62.69 (97.18)*
LN19183-450 (*n* = 60)	Pre-dose	95.84 ± 43.24	54.08±48.37
	During exercise	182.49 ± 67.67 (90.41)*	287.61 ± 185.40 (431.82)*#
	20 min post-exercise	75.22 ± 36.99 (-21.51)*#	93.11 ± 75.95 (72.17)*
		Serum Free Fatty acids, μM (% Change from Day 1)	
Placebo (*n* = 60)	Day 1	134.50 ± 86.91	
	Day 2	146.71 ± 83.21 (9.07)	
LN19183-450 (*n* = 60)	Day 1	138.91 ± 70.50	
	Day 2	177.00 ± 84.04 (27.42)*#	

Data present as mean ± SD.

* indicates significance (*P* < 0.05) in intragroup comparison (vs. pre-dose) analyzed using paired *t*-test.

# indicates significance (*P* < 0.05) in intergroup comparison (vs. placebo) analyzed using a mixed model considering treatment, period, and sequence as fixed factors.

LN19183 administration increased the serum levels of free fatty acids (FFA) on day 2. This change was significant as compared to day 1 (*P* < 0.0001) and placebo (*P* = 0.0221) (Table 2). However, these values are within the normal ranges (25).

### LN19183 supplementation minimally alters the participants’ hemodynamic variables

Table 3 summarizes the systolic and diastolic blood pressure (SBP and DBP, respectively) and heart rates (HR) of the LN19183-supplemented participants at rest and with exercise. The SBP, DBP, and HR of the placebo and LN19183 groups were comparable on days 1 and 2. Between-the-groups comparison analyses reveal that the values are not significantly different, except the HR at 120 min on day 1 (at rest) and at 20 min post-exercise on day 2 in the LN19183-supplemented participants (Table 3). However, these changes were within the normal ranges.

**Table 3:** Effect of LN19183 supplementation on participants’ blood pressure and heart rate

Interventions	Measures	Evaluations at			
		Pre-dose	60 min	120 min	180 min
Day 1					
Placebo (*n* = 60)	Systolic BP	115± 5	116 ± 6*	117 ± 5*	119 ± 6*
	Diastolic BP	71 ±5	73 ± 5*	73 ± 6*	74 ± 6*
	Heart Rate	70 ± 7	69 ± 6	69 ± 7	70 ± 7
LN19183-450 (*n* = 60)	Systolic BP	114 ± 5	116 ± 6*	118 ± 6*	119 ± 5*
	Diastolic BP	72 ± 5	74 ± 6*	73 ± 5*	74 ± 5*
	Heart Rate	69 ± 7	69 ± 6	71 ± 8*#	71 ± 8*
Day 2					
		Pre-dose	During exercise	20 min post-exercise	
Placebo (*n* = 60)	Systolic BP	115 ± 5	147 ± 5*	119 ± 5*	
	Diastolic BP	71 ± 4	73 ± 5*	73 ± 4*	
	Heart Rate	71 ± 7	139 ± 10*	75 ± 8*	
LN19183-450 (*n* = 60)	Systolic BP	115 ± 5	146 ± 6*	118 ± 4*	
	Diastolic BP	72 ± 4	73 ± 4*	74 ± 4*	
	Heart Rate	71 ±7	136 ± 12*	78 ± 10*#	

Data present as mean ± SD of the systolic and diastolic blood pressure (BP) in mmHg and heart rate in beats/min.

* indicates significance (*P* < 0.05) in intragroup comparison (vs. pre-dose) analyzed using paired *t*-test.

# indicates significance (*P* < 0.05) in intergroup comparison (vs. placebo) analyzed using a mixed model considering treatment, period, and sequence as fixed factors.

### LN19183 supplementation reduced participants’ mood disturbance scores

On day 2, the LN19183-supplemented participants showed reduced total mood disturbance scores (22.80%, *P* < 0.0001 vs. day 1; 19.33%, *P* = 0.0167 vs. placebo) (Supplementary Table 2). The placebo group also showed a 9.91% (*P* = 0.0672 vs. day 1) lower mood disturbance scores on day 2.

### LN19183 did not alter serum clinical chemistry and hematology parameters

The observations on the serum clinical chemistry and hematology parameters in the placebo and LN19183-supplemented participants are presented in Supplementary Table 3. The values at the screening visit and the end of the study (day 14) in the placebo and LN19183 groups did not alter significantly and remained within the normal ranges (Supplementary Table 3). The participants did not report any adverse events during the study.

## Discussion

In general, the subjects with higher metabolic rates have an overall superior metabolic profile and anthropometric measures as compared to the obese or overweight individuals with lower metabolic rates. Previous studies established that ingesting thermogenic supplements containing caffeine in combination with various ingredients can acutely increase RMR (26, 27). On the contrary, selected studies suggest that thermogenic products effectively augment energy expenditure but raise a general concern about their impact on hemodynamic variables, causing tachycardia, heart palpitations, anxiety, and headaches (28). Recently, Kundimi et al. demonstrated that a synergistic composition of *C. aurantifolia* fruit rind and *T. cacao* seed extracts (LN19183), a negligible source of caffeine, increased REE via activation of FGF-21/β3-AR/UCP-1 axis, enhanced fat oxidation, and reduced adiposity in HFD-fed obese rats (13). Although the plant raw materials are popular ingredients in the food, confectionery, and beverage industries worldwide, a comprehensive toxicological evaluation in OECD-approved sub-chronic repeated dose oral and genotoxicity studies confirmed a comprehensive spectrum safety of LN19183 (communicated separately). However, in continuation of the previous observation, the present preclinical study demonstrates a dose-dependent efficacy of LN19183 in increasing REE and further supports the thermogenic potential of this novel phytoceutical composition in reducing body weight and adiposity in HFD-fed obese rats. Furthermore, a cross-over clinical proof-of-concept study demonstrates an acute effect of LN19183 supplementation in increasing the metabolic rates at rest and exercise in overweight male and female subjects without adverse effects.

FGF-21 induces UCP-1 expression, which in turn triggers thermogenesis or energy expenditure in the adipose tissues (29, 30). UCP-1 upregulation in association with increased PGC-1α, β3-AR in PPARα-agonists-supplemented obese mice demonstrated enhanced thermogenesis accompanied by significant body mass reduction (31). Furthermore, the drug candidates, including butein, β-aminoisobutyric acid, and salsalate, have been shown to induce UCP-1 expression in white adipocytes and prevent weight gain in diet-induced obese mice, suggesting their possible therapeutic potentials in weight management (32). This evidence suggests that the induction of UCP-1 in WAT is a potential strategy for weight management in overweight or obese individuals. In this context, it is important to emphasize that during cold exposure, β-adrenergic stimulation activates UCP-1 to trigger thermogenesis via browning the WAT and increase lipid breakdown (33). β3-AR agonists, isoproterenol and CL316.243, are reported to increase UCP-1 expression at the mRNA and protein levels in brown and white adipocytes. Furthermore, these agonists have been shown to activate BAT and induce WAT browning in mice (34–36).

This study highlights that LN19183 increases thermogenic potential without altering blood pressure (BP) and with minimal increase in heart rate (HR) of the human volunteers. In this proof-of-concept study, the observations on these hemodynamic variables suggest that LN19183 does not stimulate the central nervous system (CNS) or possesses a sympathomimetic activity. The stimulant-based thermogenic agents that include caffeine or ephedrine exert positive inotropic effects on the heart, thus increasing HR and BP via enhanced sympathetic stimulation involving epinephrine, norepinephrine, and beta-adrenergic receptors α-, β1, and β2 (37, 38). In contrast, the non-stimulant thermogenic phytochemicals, such as p-synephrine, are reported to selectively activate β3-AR than involving epinephrine, norepinephrine, or other β-ARs, thus reducing the cardiovascular adverse effects (39). Other thermogenic phytonutrients such as forskolin, chlorogenic acid, or carotenoids have been reported to enhance energy expenditure via AMPK activation or mitochondrial UCP-1 induction (39). In this clinical study, LN19183 administration significantly increased metabolic rates at rest and with exercise in the volunteers. The increases in energy metabolism associated with the enhanced rates of fat and carbohydrate oxidation in the human volunteers corroborate with the thermogenic efficacy of LN19183 in the present and earlier published rat studies (13). In this context, the increased phosphorylation of the master metabolic regulator AMPK and the thermogenic marker proteins in the liver and fat tissues are interesting, and these events may partially explain the biochemical basis of thermogenic efficacy of LN19183 via modulating the energy metabolism in the human volunteers.

The present preclinical observations are consistent with the previous investigation (13). LN19183 supplementation activated the FGF-21/β3-AR/UCP-1 axis to increase REE in association with fat and carbohydrate oxidation, reduced body weight gain and adiposity in the HFD-fed obese rats, and improved adiponectin and leptin levels. Furthermore, the present rat study has demonstrated that LN19183 supplementation increased AMPK activation. The AMPK-ACC-malonyl CoA signaling cascade plays a major regulatory role in activating carnitine palmitoyl transferase-alpha (CPT-1α) to stimulate fatty acid oxidation (40). The upregulation of UCP-1 and β3-AR is essential for thermogenesis (41). β3-AR activation is a potential pharmacological approach for stimulating brown and beige fat, and a β3-AR agonist has been demonstrated to be effective in activating BAT, resting energy expenditure, and inducing beige fat cells in humans (41–43). Furthermore, a significant role of GLP-1 in thermogenesis via WAT to BAT transformation has been established in preclinical and clinical studies, and the use of GLP-1 receptor agonists has emerged as an attractive therapeutic approach for the managing body weight and healthy blood sugar levels (44–46). In this context, the increased GLP-1R protein expression in the LN19183-supplemented rat liver is quite interesting, supporting the thermogenic and body weight reduction efficacy of LN19183 in rats. In continuation of this research, further exploration of the effect of LN19183 on circulatory levels of GLP-1 or DPP-IV inhibition would be attractive. However, in this study, increased AMPK activation associated with CPT-1α expression and increased protein expressions of other thermogenic factors such as β3-AR, FGF-21, and UCP-1 in the fat tissue suggest and corroborate the earlier observations on thermogenic potential and further elucidate a possible basis of a transformation of white fat into brown fat in LN19183-supplemented rats (13).

This study has a few limitations. In preclinical research, studying more beige- or BAT-specific markers at protein and gene levels would have provided a more comprehensive mechanism of action for LN19183, supporting its thermogenic role via activating WAT to transform into BAT. This study did not measure the effect of LN19183 on insulin sensitivity in the HFD-fed obese rats. However, the increased serum adiponectin and liver GLP-1R expression and reduced serum glucose levels suggest a possible role in improving insulin sensitivity in obese rats. The clinical study is short term and is intended to evaluate the acute effect of LN19183. Although the individual plant materials have been consumed widely for ages, this clinical study does not provide enough support for the long-term and comprehensive safety of LN19183 composition.

Understanding the mechanism of action from the earlier study (13) and the present preclinical and proof-of-concept clinical efficacy study data, we anticipate that long-term consumption of LN19183 would result in improved body composition by reducing fat mass and managing healthy body weight in human adults. Also, LN19183 consumption may help reduce hyperlipidemia and manage healthy blood sugar levels via regulating fat and carbohydrate metabolism. Further studies are warranted to determine the long-term efficacy and safety of LN19183.

## Conclusion

In this investigation, the animal study reaffirmed the thermogenic potential of LN19183, which positively modulates the molecular markers that are involved in the white-to-brown fat transformation, such as CPT-1α and β3-AR, resulting in reduced body fat mass and weight gain in HFD-fed obese rats. Furthermore, the proof-of-concept human study demonstrates the thermogenic potential of nonstimulant LN19183 that elevated fat oxidation. Together, these observations suggest the clinical benefits of LN19183 consumption, including fat oxidation, healthy body weight, and alleviating metabolic dysfunctions.

## Supplementary Material



## Data Availability

The data supporting the published observations are available on request.
