# Supplementary material for: A combination of Citrus aurantifolia fruit rind and Theobroma cacao seed extracts supplementation enhances metabolic rates in overweight subjects: a randomized, placebo-controlled, cross-over study
Source: Food Nutr Res. 2024 Aug 1;68:10.29219/fnr.v68.10745. doi: 10.29219/fnr.v68.10745 (PMC11305151; doi:10.29219/fnr.v68.10745)
Supplement: Supplementary file 1 [file FNR-68-10745-s1.docx]

**Supplementary Table 1: Inclusion-exclusion criteria**

| **Inclusion criteria** |
| --- |
| - Healthy male and female subjects between 20-39 years with body mass index of 25 - 29.9 kg/m^2^. |
| - Subjects with a sedentary lifestyle, no regular athletic or sports activities. |
| - Normal thyroid hormone profile and electrocardiogram (ECG) |
| - Healthy as per health history and routine clinical investigations during screening. |
| - No consumption of coffee, tea, cola, energy drinks, chocolate and nutritional supplements before 24 hours of randomization and evaluation days of the study |
| - Subjects agreed to sign the written informed consent and maintained daily diaries and diet tracker. |
| - A negative pregnancy test during recruitment, no lactating mothers, and willing to use medically acceptable contraception for female participants |
| **Exclusion criteria** |
| - Loss or gain of body weight greater than 3 kg in past 3 months. |
| - Smoking and/or alcohol consumption habits |
| - Consumption of nutritional supplements (e.g., creatine, protein drinks, amino acids, or vitamins) or ergogenic aids within past 30 days and during the study. |
| - Subjects who are participating or currently participating in another clinical trial within 30 days prior to screening. |
| - Medical condition including metabolic disorders (obesity, hyperlipidemia, diabetes, hypertension, atherosclerosis), arthritis, cancer, or taking medications including, antidepressants, beta-blockers, hormones therapies, etc. |
| - Subjects underwent treatment for COVID-19 or tested positive COVID-19 or HIV during screening |

**Supplementary Table 2: POMS scores**

| Groups | Evaluation days | Total Mood Disturbance scores | % Change from baseline |
| --- | --- | --- | --- |
| Placebo  (n=60) | Day1 | 11.60 ± 5.18 | - |
|  | Day 2 | 10.45 ± 4.66 | -9.91 |
| LN19183-450 (n= 60) | Day1 | 10.92 ± 4.47 | - |
|  | Day 2 | 8.43 ± 4.79*# | -22.71 |

Data present as mean ± SD. * and # indicate significance (*p* < .05) in intragroup comparison (Day 1 vs. Day 2) and intergroup comparison (Placebo vs. LN19183) analyzed using paired t-test and two-way ANOVA, respectively

**Supplementary Table 3: Serum biochemistry and hematology parameters**

|  | Parameters | Groups | Screening | Day 14 |
| --- | --- | --- | --- | --- |
| Serum biochemistry | Glucose  (mg/dL) | Placebo/LN19183 | 86.87 ± 8.33 | 88.27 ± 7.39 |
|  |  | LN19183/Placebo | 83.99 ± 17.08 | 88.20 ± 6.38 |
|  | Creatinine  (mg/dL) | Placebo/LN19183 | 0.84 ± 0.16 | 0.86 ± 0.14 |
|  |  | LN19183/Placebo | 0.86 ± 0.18 | 0.84 ± 0.17 |
|  | Blood Urea Nitrogen  (mg/dL) | Placebo/LN19183 | 11.09 ± 3.57 | 12.53 ± 3.41 |
|  |  | LN19183/Placebo | 12.95 ± 3.61 | 13.14 ± 3.61 |
|  | Uric Acid  (mg/dL) | Placebo/LN19183 | 5.59 ± 1.57 | 5.54 ± 1.23 |
|  |  | LN19183/Placebo | 5.10 ± 1.17 | 5.30 ± 1.06 |
|  | Sodium  (mmol/L) | Placebo/LN19183 | 139.77 ± 2.65 | 139.80 ± 2.52 |
|  |  | LN19183/Placebo | 140.03 ± 3.29 | 140.27 ± 2.64 |
|  | Potassium  (mmol/L) | Placebo/LN19183 | 4.07 ± 0.31 | 4.13 ± 0.40 |
|  |  | LN19183/Placebo | 4.07 ± 0.35 | 4.27 ± 0.93 |
|  | Alanine transaminase  (IU/L) | Placebo/LN19183 | 23.99 ± 12.64 | 23.93 ± 6.34 |
|  |  | LN19183/Placebo | 24.33 ± 7.76 | 25.33 ± 6.40 |
|  | Aspartate aminotransferase  (IU/L) | Placebo/LN19183 | 25.60 ± 6.79 | 27.90 ± 5.65 |
|  |  | LN19183/Placebo | 26.67 ± 6.75 | 28.70 ± 4.96 |
|  | Alkaline phosphatase  (IU/L) | Placebo/LN19183 | 81.60 ± 22.98 | 85.13 ± 19.62 |
|  |  | LN19183/Placebo | 86.33 ± 20.55 | 92.10 ± 20.35 |
|  | Bilirubin  (mg/dL) | Placebo/LN19183 | 0.68 ± 0.45 | 0.64 ± 0.29 |
|  |  | LN19183/Placebo | 0.51 ± 0.22 | 0.58 ± 0.22 |
|  | Albumin  (g/dL) | Placebo/LN19183 | 4.63 ± 0.40 | 4.67 ± 0.48 |
|  |  | LN19183/Placebo | 4.65 ± 0.31 | 4.60 ± 0.36 |
|  | Low-density lipoprotein  (mg/dL) | Placebo/LN19183 | 92.70 ± 22.23 | 90.43 ± 20.45 |
|  |  | LN19183/Placebo | 97.29 ± 26.59 | 95.94 ± 27.09 |
|  | High-density lipoprotein  (mg/dL) | Placebo/LN19183 | 37.00 ± 7.74 | 37.20 ± 5.84 |
|  |  | LN19183/Placebo | 42.00 ± 8.69 | 42.56 ± 8.63 |
|  | Very low-density  lipoprotein (mg/dL) | Placebo/LN19183 | 24.10 ± 9.57 | 25.47 ± 9.36 |
|  |  | LN19183/Placebo | 23.21 ± 6.19 | 22.59 ± 7.49 |
|  | Triglycerides  (mg/dL) | Placebo/LN19183 | 121.33 ± 47.65 | 128.13 ± 47.07 |
|  |  | LN19183/Placebo | 115.77 ± 30.75 | 112.13 ± 36.71 |
|  | Total cholesterol  (mg/dL) | Placebo/LN19183 | 153.80 ± 23.84 | 153.10 ± 24.52 |
|  |  | LN19183/Placebo | 162.50 ± 31.46 | 161.09 ± 30.98 |
| Hematology | Hemoglobin  (g/dL) | Placebo/LN19183 | 14.42 ± 1.76 | 14.54 ± 1.74 |
|  |  | LN19183/Placebo | 14.34 ± 1.35 | 14.64 ± 1.40 |
|  | Platelet count  (10^5^ /cu.mm) | Placebo/LN19183 | 2.57 ± 0.62 | 2.63 ± 0.57 |
|  |  | LN19183/Placebo | 2.67 ± 0.56 | 2.75 ± 0.55 |
|  | Erythrocyte sedimentation rate (mm/hr) | Placebo/LN19183 | 9.13 ± 1.80 | 8.90 ± 1.35 |
|  |  | LN19183/Placebo | 9.13 ± 2.27 | 8.93 ± 1.14 |
|  | RBC count  (10^6^/cu.mm) | Placebo/LN19183 | 5.03 ± 0.42 | 5.13 ± 0.45 |
|  |  | LN19183/Placebo | 5.00 ± 0.45 | 5.04 ± 0.41 |
|  | WBC  (cells/cu.mm) | Placebo/LN19183 | 6543 ± 1504 | 6642 ± 1431 |
|  |  | LN19183/Placebo | 7407 ± 1723 | 7594 ± 1771 |
|  | Neutrophil (%) | Placebo/LN19183 | 53.26 ± 7.68 | 55.84 ± 6.04 |
|  |  | LN19183/Placebo | 54.01 ± 6.40 | 56.93 ±6.39 |
|  | Lymphocytes (%) | Placebo/LN19183 | 36.52 ± 7.12 | 34.00 ±6.41 |
|  |  | LN19183/Placebo | 36.13 ± 6.01 | 33.02 ±5.91 |
|  | Eosinophil (%) | Placebo/LN19183 | 3.54 ± 1.76 | 3.55 ± 1.42 |
|  |  | LN19183/Placebo | 3.21 ± 1.13 | 3.54 ± 1.75 |
|  | Monocytes (%) | Placebo/LN19183 | 6.03 ± 1.26 | 6.04 ± 1.23 |
|  |  | LN19183/Placebo | 6.08 ± 1.36 | 5.94 ± 1.40 |
|  | Basophils (%) | Placebo/LN19183 | 0.66 ± 0.52 | 0.56 ± 0.19 |
|  |  | LN19183/Placebo | 0.58 ± 0.25 | 0.58 ± 0.25 |
